# Effects of Particle Migration on the Relaxation of Shock Wave Collisions

**DOI:** 10.3390/e26090724

**Published:** 2024-08-25

**Authors:** Hao Li, Bo Xu, Zixiang Yan, Xinyu Zhang, Chongjie Mo, Quanxi Xue, Xiazi Xiao, Hao Liu

**Affiliations:** 1 Department of Applied Physics, School of Physics and Electronics, Hunan University, Changsha 410082, China; 2 School of Science, Beijing University of Posts and Telecommunications, Beijing 100876, China; 3 Center for Applied Physics and Technology, and College of Engineering, Peking University, Beijing 100871, China; 4 Beijing Computational Science Research Center, Beijing 100193, China; 5 State Key Laboratory of Laser Interaction with Matter, Northwest Institute of Nuclear Technology, Xi’an 710024, China; 6 Department of Mechanics, School of Civil Engineering, Central South University, Changsha 410075, China

**Keywords:** shock wave, non-equilibrium process, relaxation process, particle migration, molecular dynamics

## Abstract

The non-equilibrium characteristics during the shock relaxation process hold a foundational position in various fields. In contrast to the propagation of a single shock wave, the collision process of two shock waves exhibits distinct non-equilibrium features. Employing non-equilibrium molecular dynamics, we simulated the collision of ultra-strong shock waves in a classical gas system, investigating the relationship between equilibrium relaxation time and shock intensity. Tracking the spatial migration of microscopic particles in the shock collision region during the relaxation process, we observed a significant contribution of particle migration to the average energy changes during relaxation. The discussion on particle migration provides a valuable new perspective for understanding the microscopic mechanisms of the relaxation process.

## 1. Introduction

Shock waves play a crucial role in diverse fields, including inertial confinement fusion (ICF) [1,2,3,4], astrophysics [5,6,7,8], hypersonic flight [9,10,11], and more [12,13,14,15]. Achieving ultra-high density and temperature for fusion fuels in ICF relies on generating superintense shock waves with powerful drivers like lasers or X-ray sources. Similarly, astrophysical investigations often require creating extreme states of matter through shock loading in labs. The non-equilibrium effects induced by shock waves significantly influence system evolution at the microscale. Understanding these dynamics is vital, especially in scenarios involving extreme states, where shock-induced non-equilibrium effects critically shape the system’s behavior.

The collision of two shock waves plays a crucial foundational role in issues such as the ICF shock ignition scheme [16,17,18] and the double cone collision ignition scheme [19]. The collision of two shock waves is a key process in elevating the density and temperature of fusion fuel. Non-equilibrium features in this shock wave collision may differ significantly from those observed in single shock wave scenarios. Current research on shock-induced non-equilibrium effects is primarily focused on individual shock waves, with limited investigation into non-equilibrium relaxation processes during the collision of two shock waves using microscopic methods.

The interaction between shock waves and matter will compress and heat the material in an extremely short period. For a stable, uniformly propagating shock wave, focusing our attention on a specific piece of material reveals that during the passage of the shock wave, the material undergoes a thermodynamic transition from one state, $S_{0}$, to another state, $S_{1}$. Consequently, there will be an equilibrium relaxation process from the $S_{0}$ state to the $S_{1}$ state. During this relaxation process, the material’s state deviates significantly from equilibrium, displaying strong non-equilibrium characteristics. Due to the steady and uniform propagation of the shock wave, when we establish the reference frame on the shock wave front and move with it, the equilibrium relaxation process experienced by the material over time will form a stable spatial distribution. This distribution can be referred to as the shock wave front structure.

The shock wave front structures operate on an extremely small spatial scale, comparable to the mean free path of molecules. The macroscopic continuum mechanics struggle to capture these structures. In continuum methods, shock waves are often depicted as infinitesimally thin discontinuities, with thermodynamic quantities interconnected through fluid conservation relations. In classical computational fluid dynamics, abrupt thermodynamic transitions across shock wave fronts cause problems like numerical oscillations, necessitating specialized treatments such as reducing computational scheme orders and introducing artificial viscosity [20]. A comprehensive understanding of shock waves necessitates a closer examination of their microscopic structure and the diverse non-equilibrium dynamic processes that occur within them.

A more accurate depiction of shock waves demands an exploration from the viewpoint of microscopic particles. Early investigations into the microscopic structure of shock wave fronts primarily employed kinetic theory methods, focusing on variations in the distribution function of microparticles under shock conditions. A pivotal theoretical contribution in this domain was the Bimodal approximation proposed by Mott-Smith [21] for the distribution function at shock wave fronts. This model subsequently laid the foundation for a series of subsequent studies [22,23,24]. Kinetic analyses are based on the Boltzmann equation, where the choice of collision models plays a crucial role in the system’s evolution. In scenarios where systems are sparsely populated and slightly deviate from equilibrium, collisional influences are minor, enabling kinetic analyses to effectively portray the evolution of distribution functions. However, for dense systems and higher degrees of non-equilibrium, the significance of collisional effects amplifies, rendering simplistic collision models inadequate in kinetic analyses.

Advancements in computer technology and molecular dynamics methods have liberated researchers from the constraints of kinetic theory, enabling more direct and detailed descriptions of particle collisions at the microscale [25,26,27]. Consequently, a series of computational studies employing the molecular dynamics method have emerged, focusing on the microscopic structure of shock wave fronts and the non-equilibrium processes induced by shock effects. Research endeavors span shock wave front thickness [28,29,30,31,32,33], distribution functions [24,34], and extend to more intricate microscopic dynamic phenomena including shock-induced phase transitions [35,36,37,38], molecular dissociation, ionization, and other complex phenomena [39,40].

In this work, we systematically simulated the collision process of strong shock waves in dense gas systems using the Non-Equilibrium Molecular Dynamics (NEMD) method. To form a clear and analyzable physical picture and eliminate the possible complex effects such as ionization, dissociation, and radiation under strong shocks, we restricted our study to a shock system that only includes a gas system with classical two-body collisions. In this paper, we conducted simulation studies using a classical helium gas system as a representative. The focus of this work is on the impact of shock wave intensity on non-equilibrium characteristics; thus, scenarios with different shock wave velocities were set. The selected range of shock wave velocities covers typical speeds observed in ICF implosion conditions.

This work will explore the relationship between the equilibrium relaxation time of various thermodynamic quantities and the shock velocity. By tracking and statistically analyzing the collision and motion of microscopic particles in the collision region of shock waves, the impact of particle collisions and migration mechanisms on the relaxation process will be examined and discussed. The statistical results indicate that as the shock velocity increases, the relaxation time monotonically decreases. Furthermore, the analysis of particle migration behavior suggests that particle migration plays a crucial role in accelerating the relaxation process.

The subsequent arrangement of this paper is outlined as follows. In Section 2, we will provide an exposition of the specific configurations of the molecular dynamics simulations and the statistical methodologies employed. Section 3 will be dedicated to the discussion of the obtained simulation results. Finally, Section 4 will encompass a comprehensive summary of the entire study.

## 2. Computational Details

### 2.1. NEMD Configurations

The non-equilibrium molecular dynamics (NEMD) calculations in this study were conducted using the LAMMPS [41] code (lammps-7Aug19). The simulated shock medium was helium, corresponding to an initial temperature of $T_{0}=20$ K (above the gas–liquid phase transition temperature of helium) and an initial density of $\rho_{0}=0.126$ g/cm^3^ (referencing parameters from Nellis’ shock experiments [42]). The system was equilibrated to the predefined initial state using the Nose–Hoover thermostat [43,44,45] and later set to the NVE ensemble in LAMMPS.

**Figure 1:**
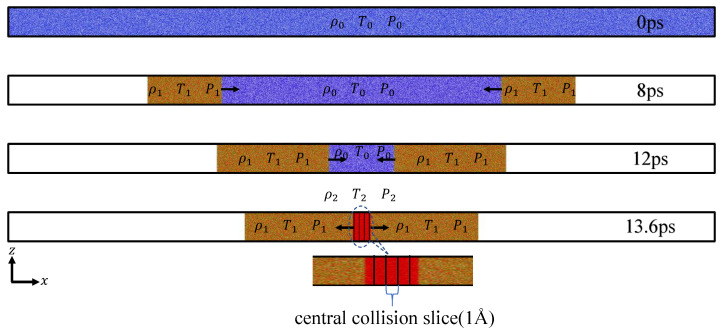
A schematic diagram of shock wave collision simulation. Shock waves are generated in dense helium by the collision of opposing reflective boundaries moving at a constant velocity on both sides. In the central collision region, uniform slices with a thickness of 1 Å are taken, and the microscopic kinetic quantities of particles within the slices are utilized to statistically determine the thermodynamic quantities corresponding to the slices.

In the calculations, the X-axis was chosen as the direction of shock wave propagation. To drive a stable, propagating pair of shock waves within the system, we established a pair of reflecting boundaries as shock pistons. These pistons move uniformly in opposite directions along the x-axis at a predetermined piston velocity $v_{p}$. To ensure sufficient evolution time for both shock waves to enter the stable propagation stage before colliding, the computational box was configured as an elongated region with dimensions of 4065 Å(x) × 203 Å(y) × 203 Å(z), containing over 2.5 million atoms, as depicted in Figure 1.

In this study, the classical Lennard-Jones (LJ) potential function is employed to describe the pairwise interactions between helium atoms, which could be written as

(1) $$V\left(r\right)=4\varepsilon \left[{\left(\frac{\sigma }{r}\right)}^{12}-{\left(\frac{\sigma }{r}\right)}^{6}\right],$$

where *r* is the distance between particles, ε is the strength of potential energy, and σ is the distance scale between particles. According to LJ potential [46], the values of ε and σ are set as ε=0.02 kcal/mol and σ=2.56 Å. As mentioned in the introduction, the subject of this study is a classical gas dominated by two-body collisions. Therefore, the application of the Lennard-Jones (LJ) potential can reveal the main physical picture. For the primary calculations, we also performed repeated simulations using the Beck [47] and Morse [48] pair potentials for helium. The comparison shows that the computational results are consistent across different potential functions.

Periodic boundary conditions were applied along the y-axis and z-axis directions to minimize the influence of lateral dimensions’ boundary effects and size effects on the propagation of shock waves [49]. The coordinate origin was set at the geometric center of the box. With the left and right pistons moving at the same velocity, the system possessed mirror symmetry about the x=0 plane. This allowed the precise determination that the collision point of the shock waves occurred within the central region of the system’s geometry.

In this work, we selected a range of piston velocities $v_{p}$ from 10 km/s to 100 km/s. As time evolves, the piston will drive two shock waves propagating in opposite directions within the system. The propagation speed of the first shock wave is denoted as $v_{s1}$, which causes the material behind the shock to move at the piston velocity, and the thermodynamic state variables of density, temperature, and pressure change to $\rho_{1}$, $T_{1}$, and $P_{1}$, respectively. When the two shock waves collide in the central region, they generate a secondary shock wave propagating outward with a speed of $v_{s2}$, leading to changes in the thermodynamic state variables to $\rho_{2}$, $T_{2}$, and $P_{2}$. Table 1 presents the thermodynamic state variables behind the shock front and shock wave speeds for both primary and secondary shocks in all cases considered in this work.

Since the chosen range of shock piston velocities for simulations spans a wide range, in order to maintain the continuity of atomic trajectories and use larger time steps Δt whenever possible to conserve computational resources, the following equation is employed to estimate the optimized time step for different shock cases,

(2) $$\Delta t=\frac{1}{40}{\left(\frac{3}{4\pi n_{i}}\right)}^{\frac{1}{3}}\sqrt{\frac{k_{B}T}{m_{I}}},$$

where $n_{i}$ and *T* represent the number density of particles and the temperature in the downstream region of the shock wave, respectively. $k_{B}$ is the Boltzmann constant, and $m_{I}$ denotes the mass of helium atoms. Although the number density $n_{i}$ and temperature *T* in the downstream region require accurate values from shock simulations, based on approximate relationships summarized in previous work [49], we can make a rough estimate of their likely ranges. In essence, the derived optimized time step Δt from the equation above is approximately 1/80 of the time taken for helium atoms to traverse the average distance between adjacent particles at their average thermal velocity. Across all simulation cases in this study, the time step ranges from 0.001 fs to 0.02 fs.

### 2.2. Thermodynamic Statistical Methodologies

The results of MD simulations provide the temporal evolution of kinematic quantities for a multitude of microscopic particles. To acquire corresponding macroscopic thermodynamic quantities and delve into the evolution of thermodynamic states during non-equilibrium relaxation processes, statistical analysis of these microscopic particles is essential. Considering the propagation of the shock wave along the x-axis, while the distribution of thermodynamic quantities is uniform along the y and z directions, the computational domain is divided into numerous thin slices along the x-axis for thermodynamic averaging, as illustrated in Figure 1. The thickness of each slice is denoted as Δx. The selection of slice thickness Δx necessitates the consideration of two interrelated factors. On one hand, the slice should be sufficiently thick to ensure an ample number of microscopic particles within this region, meeting the fundamental requirements of thermodynamic statistics. On the other hand, given the small spatial scale of the shock process, if the slice is excessively thick, the thermodynamic quantities obtained from statistics might lack locality, rendering them incapable of discerning the details of equilibrium relaxation processes. In this study, a slice thickness of 1 Å was ultimately chosen.

For a specific slice, the statistical formula for various thermodynamic quantities is as follows [32,50]:

(3) $$\begin{array}{cc} \rho (x) & =\frac{Nm_{I}}{\Omega } \end{array}$$

(4) $$\begin{array}{cc} u(x) & =\frac{1}{N}\sum_{i=1}^{N}u_{i} \end{array}$$

(5) $$\begin{array}{cc} T(x) & =\frac{m_{I}}{3Nk_{B}}\sum_{i=1}^{N}\left\{{\left[u_{i}-u\left(x\right)\right]}^{2}+{v_{i}}^{2}+{w_{i}}^{2}\right\} \end{array}$$

(6) $$\begin{array}{cc} P(x) & =\frac{Nk_{B}T}{\Omega }+\frac{1}{\Omega }\sum_{i\in \Omega }\sum_{j\neq i}{\vec{f}}_{ij}\cdot \left({\vec{r}}_{i}-{\vec{r}}_{j}\right) \end{array}$$

where ρ, *u*, *T*, and *P* represent the density, velocity, dynamic temperature, and pressure corresponding to the slice, respectively. *N* is the total number of particles in the slice, and Ω is the volume of the slice. $u_{i}$, $v_{i}$, and $w_{i}$ represent the components of the velocity vector of atom *i* along the x, y, and z directions, respectively. ${\vec{f}}_{ij}$ represents the force generated between atom *i* and atom *j* due to the interaction potential, ${\vec{r}}_{i}$ and ${\vec{r}}_{j}$ are the position vector of atom *i* and atom *j*, respectively.

In the process of non-equilibrium relaxation, significant non-equilibrium effects will be observed, and the kinetic energy of microparticles’ thermal motion exhibits noticeable anisotropy. To quantitatively describe this non-equilibrium characteristic, based on the fundamental principles defined by dynamic temperature, we further decompose the dynamic temperature into temperature components, denoted as $T_{\|}$ ($T_{x}$) and $T_{⊥}$ (the average of $T_{y}$ and $T_{z}$), parallel and perpendicular to the direction of the shock, respectively. The corresponding formula for $T_{\|}$ and $T_{⊥}$ can be written as [49]

(7) $$\begin{array}{cc} T_{\|} & =\frac{m_{I}}{Nk_{B}}\sum_{i=1}^{N}{\left[u_{i}-u\left(x\right)\right]}^{2} \end{array}$$

(8) $$\begin{array}{cc} T_{⊥} & =\frac{m_{I}}{2Nk_{B}}\sum_{i=1}^{N}\left({v_{i}}^{2}+{w_{i}}^{2}\right) \end{array}$$

It is worth noting that the concept of temperature requires a basis in thermodynamic equilibrium. In the non-equilibrium region of a shock wave, the ‘temperature’ and its components obtained from Equations (5), (7), and (8) cannot be directly interpreted as the temperature concept in equilibrium states. Instead, they manifest as parameters describing the characteristics of particle distribution, reflecting the deviation from Maxwell distribution under non-equilibrium conditions.

## 3. Results and Discussion

### 3.1. Overshoot Structure and Velocity Distribution

The shock wave front is notably characterized by its non-equilibrium nature, reflected on a microscopic level through deviations in the distribution function from the Maxwellian distribution. This non-equilibrium behavior extends to macroscopic thermodynamic quantities, such as the overshoot phenomenon in dynamic temperature distribution. Previous studies have extensively explored these aspects at individual shock wave fronts [24,31,34,49].

**Figure 2:**
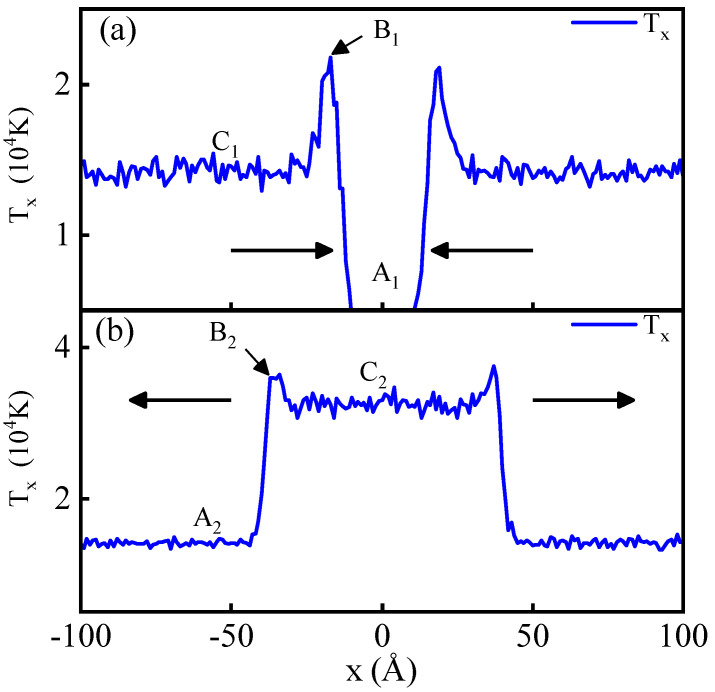
The profile of kinetic temperature $T_{x}$ along axis x before (**a**) and after (**b**) the shock collision with $v_{p}=10$ km/s. $A_{1}$, $B_{1}$, and $C_{1}$ correspond to the shock wave’s pre-shock region, overshoot at the shock front, and post-shock region during the first shock, respectively. $A_{2}$, $B_{2}$, and $C_{2}$ correspond to the respective positions during the second shock.

In this study, we further investigate non-equilibrium features in shock wave collisions by examining the evolution of distribution functions and the occurrence of the overshoot phenomenon during the collision of two shock waves. Building upon prior research, we focus on the most pronounced overshoot feature observed in the dynamic temperature component along the shock direction, denoted as $T_{x}$. To illustrate, we analyze a case with a shock piston velocity of 10 km/s, presenting distribution plots of $T_{x}$ before and after the shock wave collision (see Figure 2).

**Figure 3:**
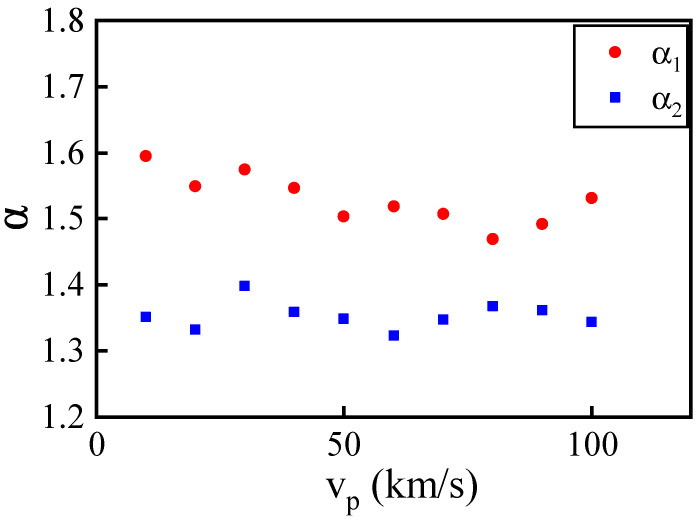
The dimensionless parameter $\alpha =T_{x}^{max}/T_{post}$ for single ($\alpha_{1}$) and secondary ($\alpha_{2}$) shocks under different $v_{p}$.

The overshoot of $T_{x}$ is manifested as a peak structure in its spatial distribution profile, as illustrated in Figure 2. Notably, the peak structure caused by a single shock wave before collision is more pronounced than the one induced by the secondary shock wave after the collision. Following the methodology of prior work [49], we quantify the overshoot using the dimensionless parameter $\alpha =T_{x}^{max}/T_{post}$, representing the ratio of the peak value $T_{x}^{max}$ to the post-wave average value $T_{post}$. Figure 3 presents the values of α at the shock wave front for both single ($\alpha_{1}$) and secondary ($\alpha_{2}$) shock waves at different shock piston velocities, taking the situation illustrated in Figure 2 as an example, where $\alpha_{1}=T_{x}^{B_{1}}/T_{x}^{C_{1}}$ and $\alpha_{2}=T_{x}^{B_{2}}/T_{x}^{C_{2}}$. In the case of a single shock wave, the results align with previous research [49], with $\alpha_{1}$ fluctuating within a small range around 1.5. However, in the case of the secondary shock wave after collision, $\alpha_{2}$ varies within a small range near 1.33, significantly lower than the single shock wave scenario.

The primary reason for this difference lies in the substantial thermodynamic differences between the shock front and the post-wave state during a single shock wave, with a density compression ratio close to 4 and a temperature increase by a factor of several thousand. In contrast, for the secondary shock wave after collision, the thermodynamic differences between the post-wave and pre-wave states decrease, with a density ratio of approximately 2 and a temperature ratio of about 4.

The overshoot phenomenon arises from the system undergoing a relaxation process, mirroring the evolution of the microparticle distribution function. We delve into this process by examining the particle velocity distribution function under shock waves, comparing functions at the overshoot peak and in the post-wave equilibrium region to understand the origins of the overshoot.

**Figure 4:**
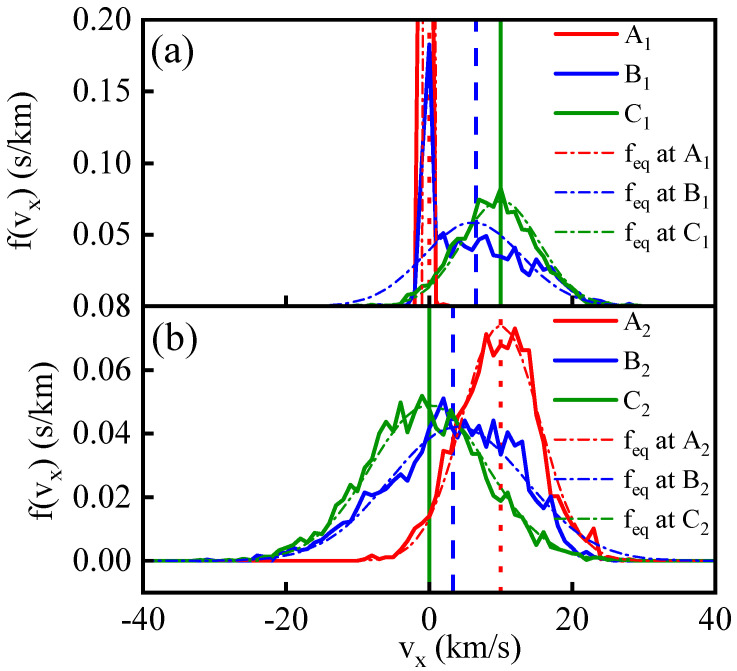
Subfigures (**a**,**b**), respectively, display the velocity distribution of $v_{x}$ at different position during the first and second shocks with $v_{p}=10$ km/s. The curves are shown at the shock front positions ($A_{1}$ and $A_{2}$), overshoot locations ($B_{1}$ and $B_{2}$), and behind the shock positions ($C_{1}$ and $C_{2}$). The specific positions corresponding to these symbols are indicated in Figure 2. The corresponding vertical lines represent the mean of the velocity distribution functions, which physically signify the macroscopic translational velocity. The curves labeled with $f_{eq}$ are the corresponding equilibrium velocity distribution.

In Figure 4, panel (a) depicts velocity distribution function curves for $v_{x}$ at various positions under a single shock wave. The vertical lines correspond to average velocity values for the colored distribution functions. At the overshoot peak, the distribution function displays a high, narrow, low-temperature peak and a broad, high-energy tail, with the average velocity notably deviating from the low-temperature peak. The calculation formula for dynamic temperature Equation (5) indicates that this “low-temperature” particle portion significantly contributes to the overall temperature after accounting for translational velocity. Contrastingly, in panel (b), the distribution function at the overshoot peak on the shock wave front after the shock wave collision is closer to the Maxwell velocity distribution curve and the average velocity falls within the central peak region of the velocity distribution function. This results in a weaker overshoot effect compared to the single shock wave scenario.

**Figure 5:**
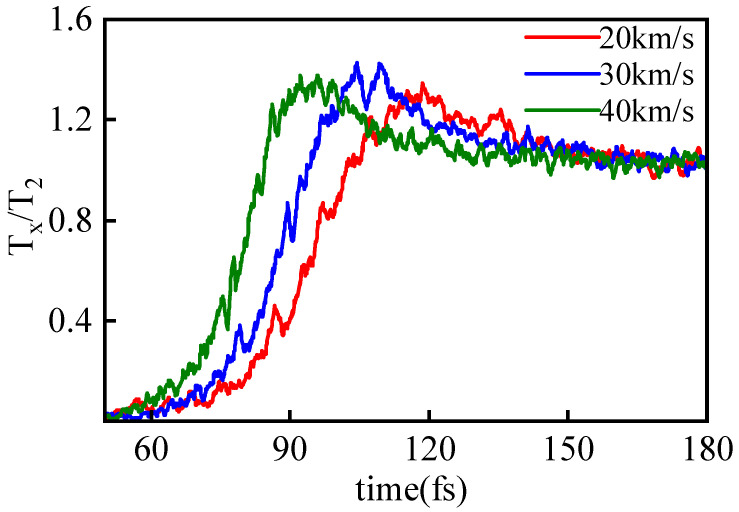
The time evolution curves of $T_{x}$ of the collision slice under different piston velocities $v_{p}$. For ease of comparison, non-dimensionalization of $T_{x}$ is performed using the equilibrium temperature after shock wave collision $T_{p}$.

The discussed overshoot phenomenon in temperature is not only observable in spatial distribution at a given moment but also in the temporal evolution within a specified region. Figure 5 illustrates the evolution of $T_{x}$ in the collision slice (−0.5 Å <x<0.5 Å) over time for piston speeds $v_{p}$ ranging from 20 to 40 km/s. The curves are normalized using the downstream equilibrium temperature for ease of comparison, revealing that the overshoot proportion remains relatively consistent across different piston speeds, while the equilibrium relaxation time decreases with higher shock intensity.

**Figure 6:**
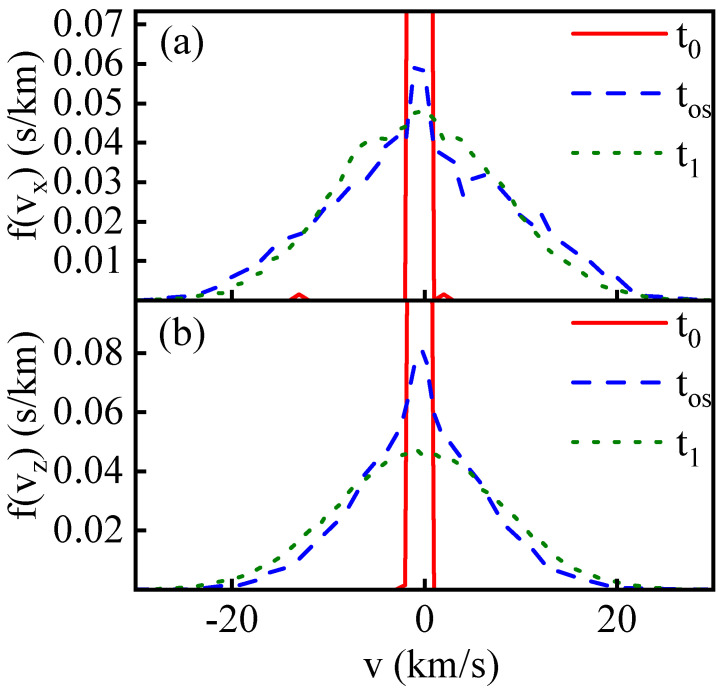
The particle velocity distribution functions at different moments within the central impact collision slice are depicted in subfigures (**a**) and (**b**) with $v_{p}=10$ km/s, corresponding to velocity components parallel ($v_{x}$) and perpendicular ($v_{z}$) to the shock direction, respectively. $t_{0}$, $t_{os}$, and $t_{1}$ correspond to the starting point of the relaxation process, the overshoot moment, and the end of the relaxation process, respectively.

In Figure 6, the blue dashed line depicts the particle velocity distribution function at the overshoot moment $t_{OS}$ of $T_{x}$ within the collision region. The green dotted line represents the particle velocity distribution function at equilibrium after the collision at time $t_{1}$, with (a) and (b) corresponding to the velocity distribution functions of $v_{x}$ and $v_{z}$, respectively.

Firstly, in comparison to the equilibrium distribution function after the collision, the velocity distribution functions of $v_{x}$ and $v_{z}$ at the moment $t_{OS}$ exhibit distinct low-temperature peak structures at v = 0 compared to the Maxwell distribution function at time $t_{1}$. This suggests the presence of numerous low-temperature particles in the region at $t_{OS}$. Meanwhile, in Figure 6a, the blue curve on both sides in the high-speed range is higher than the green dotted line. This phenomenon is attributed to high-temperature particles behind shock waves carrying not only substantial thermal kinetic energy but also significant translational kinetic energy along the shock direction. At $t_{OS}$, this translational kinetic energy is primarily retained in the x-direction, resulting in a peak in the statistical analysis of the $T_{x}$ component. As the relaxation process unfolds, this translational kinetic energy gradually disperses uniformly in all three directions through particle collisions and scattering, ultimately reaching isotropic thermodynamic temperature. Consequently, $T_{x}$ decreases from its peak value.

In contrast, in Figure 6b, the transverse velocity distribution function in the high-speed range on both sides shows that the blue dashed line is lower than the green dotted line. There is no overshoot structure in the statistically obtained $T_{⊥}$ since there is no translational kinetic energy induced by the shock in the transverse direction.

### 3.2. Relaxation Time under Shock Collision

This work primarily investigates relaxation processes in shock wave collisions. Notably, the shock system undergoes distinct thermodynamic state changes: the first shock ($S_{0}$ to $S_{1}$) and the second shock ($S_{1}$ to $S_{2}$). A variable period of equilibrium at $S_{1}$ occurs between these shocks in most regions. However, at the central collision region, the transition is direct from $S_{0}$ to $S_{2}$, causing notable differences in the equilibrium relaxation process compared to a single shock wave case. Our analysis will focus on the temporal evolution of thermodynamic quantities during the shock collision, exploring their interrelationships and connections to shock intensity.

**Figure 7:**
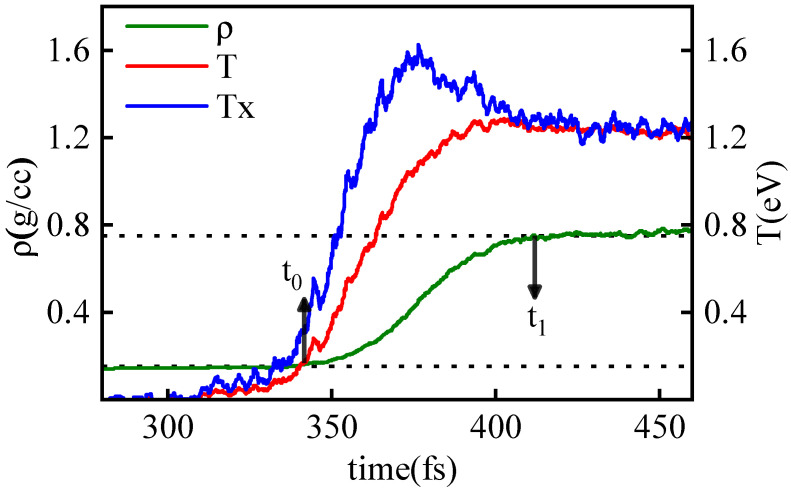
The time evolution curve of density ρ (green), temperature *T* (red), and the temperature component $T_{x}$ (blue) at the shock collision slice during the relaxation process, with $v_{p}$ = 20 km/s. The points corresponding to the deviation of density from the equilibrium values before and after the collision are marked as the starting ($t_{0}$) and ending ($t_{1}$) points of the relaxation process of density, respectively.

Figure 7 illustrates the time evolution of density (ρ), temperature (*T*), and parallel temperature components ($T_{x}$) in the shock collision region, with a piston velocity ($v_{p}$) of 20 km/s. Similar to the single shock scenario, the density curve lags behind the temperature increase, indicating that shock heating precedes shock compression. Due to distinct relaxation processes under shock, different thermodynamic quantities exhibit varying relaxation times.

Here, we obtain the relaxation time τ using the method illustrated in Figure 7. For example, the relaxation time ($\tau_{\rho }$) for density ρ is determined by considering the average density values before and after collision as reference points. Horizontal lines are drawn at these equilibrium states, and the deviation points on the density curve mark the start ($t_{0}$) and end ($t_{1}$) of the relaxation process, with the time difference representing $\tau_{\rho }=t_{1}-t_{0}$. This study conducts a statistical analysis of relaxation processes for density, temperature, and its components at different shock velocities, presenting the obtained relaxation times in Table 2.

**Figure 8:**
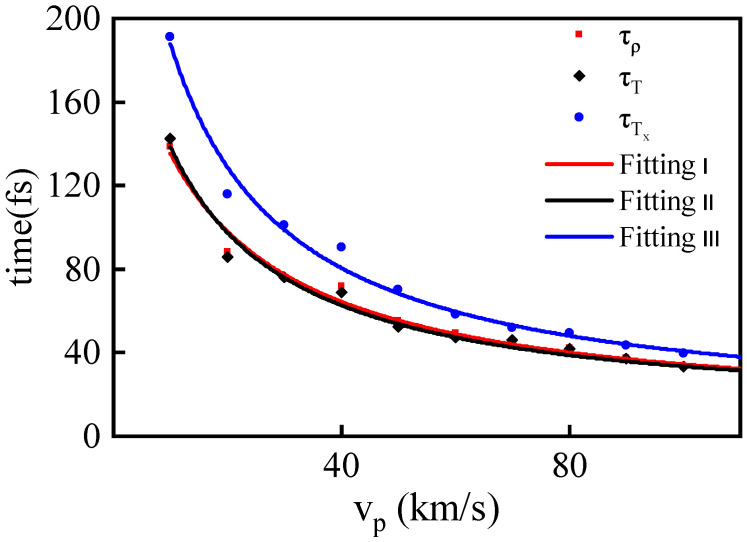
The relaxation times of density ρ (red cube), temperature *T* (black diamond), and $T_{x}$ (blue circle) under different $v_{p}$ measured in MD simulations along with the fitted curves obtained using Equation (9).

To visually depict the variation in relaxation times with shock piston velocity, we generated plots for the data. It is evident that with increasing shock intensity, the relaxation times of various thermodynamic parameters consistently decrease, as shown in Figure 8. Analyzing the decreasing trend in the $v_{p}$-τ curve, it adheres to the characteristics of an inverse proportionality function. If the hypothesis of this inverse proportional function holds true, then the relationship between τ and $v_{p}$ can be explained as follows: the thickness of the shock wave collision region remains constant, and the propagation speed of the shock wave is approximately linearly related to $v_{p}$. In this case, the relaxation time τ can be approximated as the time it takes for the shock wave to traverse this fixed thickness or is linearly related to this time.

To further clarify the relationship between relaxation times (τ) and shock piston velocity ($v_{p}$), we initially attempted a direct fit of the data from Table 2 using the inverse proportionality function y=a/x. However, the results deviated significantly from the MD simulation data points. This deviation indicates that the determination of the relaxation time for shock wave collisions cannot be explained by the simplified physical picture mentioned above alone. It necessitates consideration of the influences of more complex effects.

To improve fitting accuracy, we introduced a corrective term to the inverse proportionality function, resulting in the following fitting equation:

(9) $$y=\frac{1}{a+bx+cx^{2}}$$

where *a*, *b*, and *c* represent the fitting parameters. The final fitting outcomes closely align with the MD simulation results, as depicted in Figure 8. The values of the ultimate fitting parameters, *a*, *b*, and *c*, for each thermodynamic parameter are provided in Table 3.

### 3.3. Particle Migration During Relaxation

**Figure 9:**
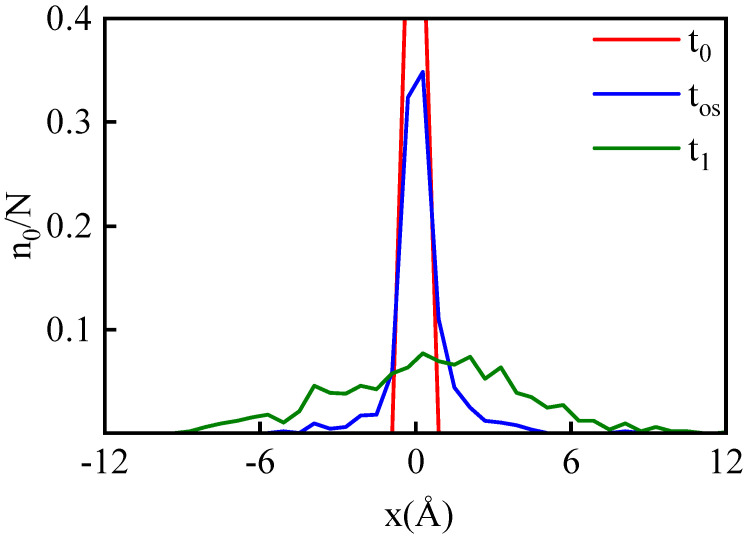
The probability distribution of G_0_ particles with $v_{p}=$ 10 km/s. The red, blue, and green curves represent the probability distribution functions of G_0_ particles at $t_{0}$, $t_{os}$, and $t_{1}$, respectively. The system is sliced along the x-direction with a thickness of 1 Å. The number of G_0_ particles in each slice $n_{0}$ is then counted.

**Figure 10:**
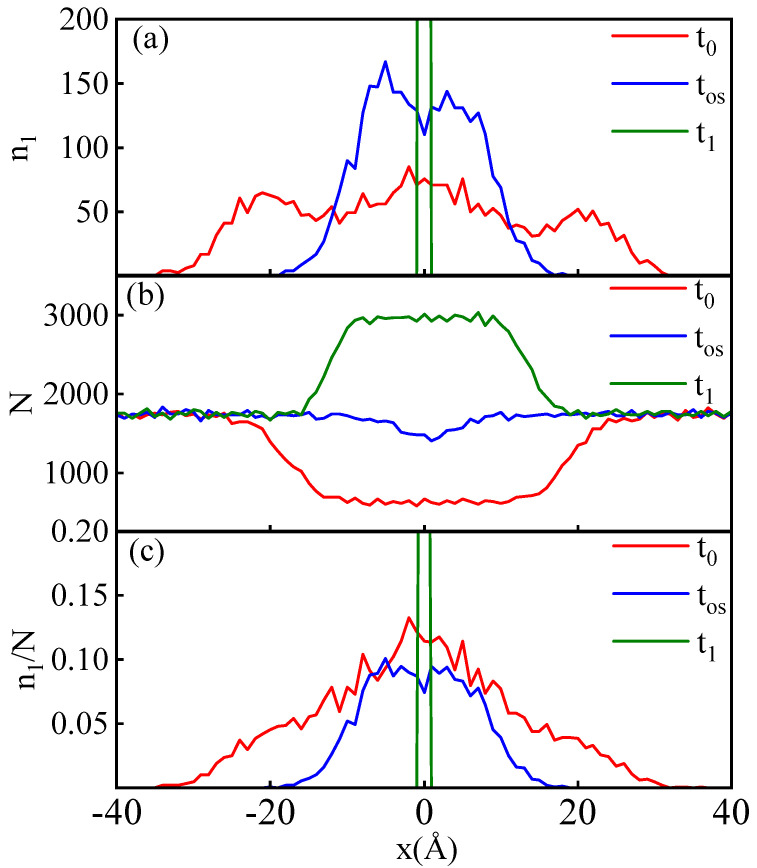
Subfigure (**a**) illustrates the particle number distribution of G_1_ within each slice. Subfigure (**b**) represents the total particle number distribution within each slice. Subfigure (**c**) displays the proportion of G_1_ particles to the total particle number within each slice $n_{1}/N$. $t_{0}$, tos, and $t_{2}$ correspond to the moments before the collision, the overshoot time, and after the collision, respectively. The corresponding $v_{p}=10$ km/s.

**Figure 11:**
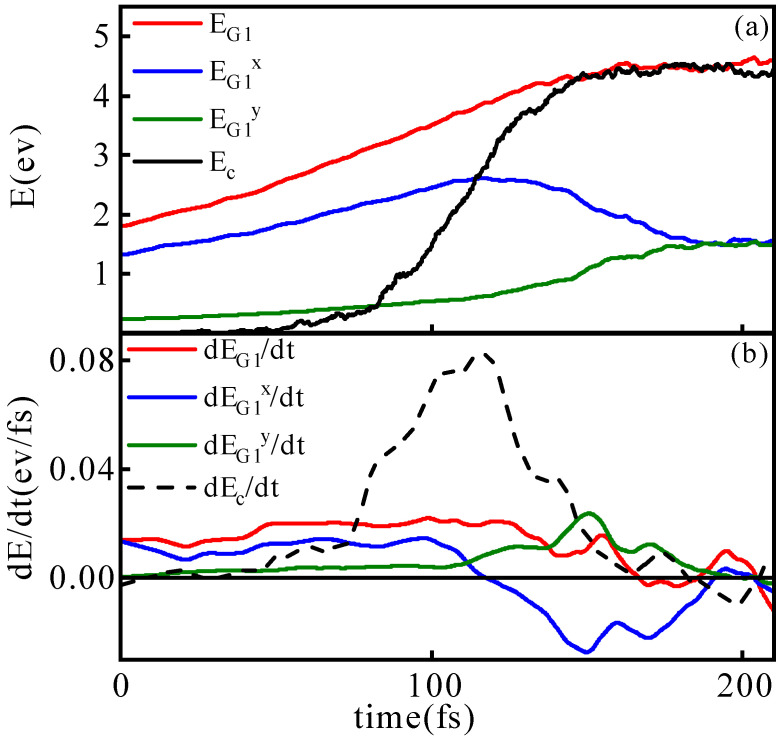
Subfigure (**a**) depicts the time evolution curve of the average kinetic energy of G_1_ particles and particles within the collision slice. Here, $E_{G1}$ represents the average kinetic energy of G_1_ particles, $E_{c}$ represents the average kinetic energy of particles within the central collision slice, and $E_{G1}^{x}$ and $E_{G1}^{y}$ represent the components of the average kinetic energy of G_1_ particles in the x and y directions, respectively. Subfigure (**b**) shows the time rate of change curves for the average kinetic energy of various particle types, as presented in Subfigure (**a**). The corresponding $v_{p}=10$ km/s.

**Figure 12:**
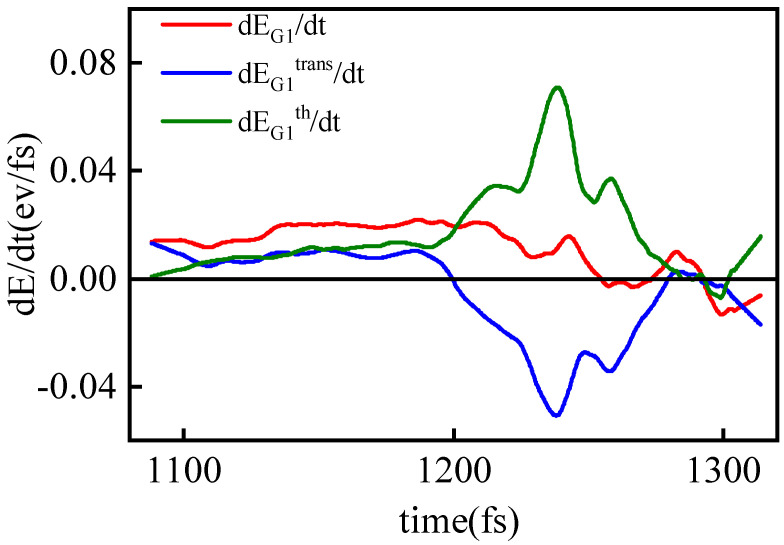
The red line depicts the time evolution rate of $E_{G_{1}}$. The blue and green curves represent the time-dependent rate of change of the translational kinetic energy component $E_{G1}^{trans}$ and the thermal kinetic energy component $E_{G1}^{th}$ of G_1_ particles, respectively. The corresponding $v_{p}=10$ km/s.

**Figure 13:**
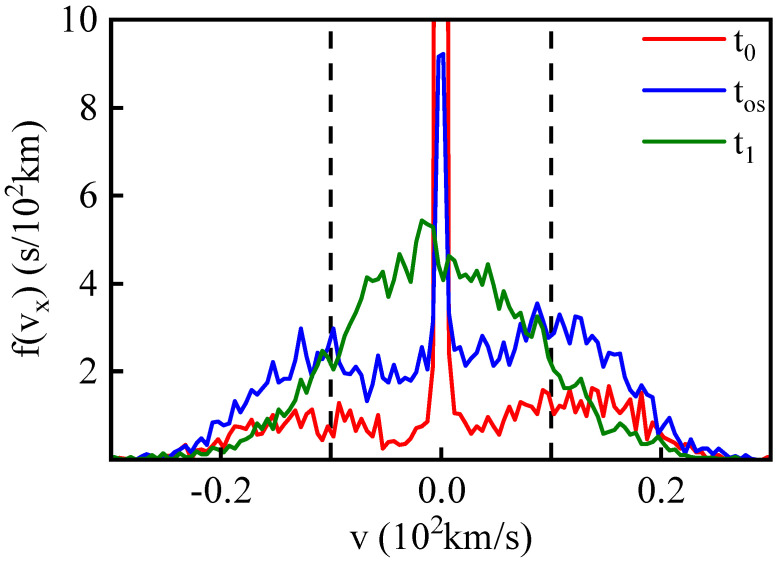
The particle velocity distribution function $f(v_{x})$ of G_1_ particles with $v_{p}=10$ km/s. $t_{0}$, tos, and $t_{2}$ correspond to the moments before the collision, the overshoot time, and after the collision, respectively. The black dashed vertical lines indicate positions corresponding to velocities of 10 km/s, which correspond to the translational velocity in the post-shock region the first shock. The distribution functions at times $t_{0}$ and $t_{os}$ in the figure show a peak structure at 10 km/s, indicating that some particles have a relatively high translational velocity.

In the following discussion, we focus primarily on two aspects. First, we explore the spatial migration patterns of particles during the relaxation process, with the relevant results shown in Figure 9 and Figure 10. Next, we examine the influence of particle migration on the energy changes of particles within the shock collision slice, with the corresponding results presented in Figure 11, Figure 12 and Figure 13.

To simplify the discussion, the following symbols will be used in the subsequent discourse. Before delving into the discussion, below is an explanation of the specific meanings associated with the symbols:

- $t_{0}$: the starting point of the relaxation process.
- $t_{1}$: the ending point of the relaxation process.
- $t_{os}$: the moment when $T_{x}$ reaches the peak of the overshoot.
- G_0_ or G_1_: the group of particles located within the collision slice at time $t_{0}$ or $t_{1}$.
- $n_{0}$ or $n_{1}$: the number of G_0_ or G_1_ particles in a slice.
- *N*: the total number of all particles in a slice.

During the relaxation process, changes in macroscopic thermodynamic quantities within the collision slice correspond to variations in microscopic particle kinematics, a transformation typically accomplished through particle collisions. However, it is crucial to note that particles in G_0_ do not remain confined to the collision slice throughout the entire relaxation process; they disperse into a broader space over time. Concurrently, particles outside the collision slice at time $t_{0}$ may also enter the collision slice during relaxation, contributing significantly to the thermodynamic state change of this slice. In addition to the contribution from particle collisions, the entry and exit of these particles play a notable role in the transformation of the thermodynamic state of the collision slice.

In the context of thermodynamic equilibrium, the movement of particles in and out of a region is primarily governed by random thermal motion, leading to particle diffusion. However, in shock wave collision systems, particles near the collision region undergo not only random thermal motion but also translational acceleration along the shock direction due to the shock effect. In subsequent discussions, we term this spatial particle change during the relaxation process as the “migration” process.

Firstly, by tracking the spatial positions of particles in G_0_, we can obtain the temporal evolution of the spatial distribution function of these particles, as shown in Figure 9. It can be observed that, for a case with $v_{p}=$ 10 km/s, within a relaxation time of 226 fs, the spatial distribution of particles in G_0_ evolves from a uniform distribution within a thin slice of thickness 1 Å to a Gaussian distribution with a half-width of approximately 7.9 Å. By comparing the results of cases with different $v_{p}$, it can be observed that with an increase in shock intensity, the spatial distribution of particles at time $t_{1}$ will broaden to a larger range.

Similar to the above analysis, we incorporate particles within the slice at $t_{1}$ into the group G_1_ and attempt to explore the contributions of particle collisions and migration to the relaxation process by tracking the spatial positions and kinematic changes of these particles in the reverse direction of time. Figure 10a displays the spatial distribution function curves of G_1_ particles at different times during the relaxation process. Unlike the situation with G_0_, the spatial distribution function at time $t_{0}$ for G_1_ does not exhibit a Gaussian distribution but instead shows three distinct peak structures, as illustrated by the red curve in Figure 10a. The peak at x=0 is due to the same reasons that lead to the peak structure of the Gaussian distribution in G_0_. Under random walk motion, the probability of a particle remaining in place at the ending time is always the highest.

The peaks on the left and right sides at time $t_{0}$ are mainly due to the non-uniformity of the particle number density in space at time $t_{0}$. At time $t_{0}$, the two shock waves have not yet fully collided; behind the shock wave front corresponds to the compressed high-density region, while the central slice, because the shock waves have not yet arrived, is in a low-density state. Therefore, the spatial profile of the particle number density will form a trough at the central slice at time $t_{0}$, as shown in Figure 10b. Despite the fact that, according to the random walk model, particles farther from the collision slice at $t_{0}$ have a lower probability of migrating to the collision slice at $t_{1}$, the higher particle density in the compressed regions on both sides ultimately leads to an increased number of particles migrating into the collision slice.

We calculate the probability of individual particles at different *x* at $t_{0}$ migrating to the central collision slice at $t_{1}$ by dividing the number of G_1_ particles within the slice at position *x* at $t_{0}$ by the total number of particles in the slice. This probability distribution with respect to spatial location is shown in Figure 10c. The probability distribution closely resembles a Gaussian curve, indicating that, from the perspective of time reversal, the particle migration process in the central collision slice during relaxation exhibits characteristics consistent with random walk motion [51], despite substantial changes in the macroscopic thermodynamic state of these particles throughout the relaxation process.

### 3.4. Changes in Particle Kinetic Energy during the Relaxation Process

The following provides explanations for the symbols that will be used in the upcoming discussion:

- $E_{G0}$ or $E_{G1}$: the average kinetic energy of particles in G_0_ or G_1_.
- $E_{G0}^{x}$ or $E_{G1}^{x}$: the component of average kinetic energy along axis *x* (the parallel direction) for particles in G_0_ or G_1_.
- $E_{G0}^{y}$ or $E_{G1}^{y}$: the component of average kinetic energy along axis *y* (the perpendicular direction) for particles in G_0_ or G_1_.
- $E_{c}$: the average kinetic energy of particles located within the collision slice.

Next, we aim to analyze the impact of particle collisions and migration on the relaxation process by examining changes in particle kinetic energy. In Figure 11a, the time evolution curves of $E_{G1}$ (red curve) and its directional components $E_{G1}^{x}$ (blue curve) and $E_{G1}^{y}$ (green curve) from $t_{0}$ to $t_{1}$ are presented, alongside the time evolution curve of $E_{c}$ (black curve).

The trend of $E_{G1}$ reveals a continuous increase in the kinetic energy of G_1_ particles throughout the relaxation process, achieved through collisions with other particles in the spatial region. The blue curve representing $E_{G1}^{x}$ initially increases and then decreases during relaxation. This behavior is primarily due to the presence of some G_1_ particles in the post-shock wave region at $t_{0}$, carrying both thermal and translational kinetic energy in the shock direction. As the relaxation progresses, collisions between particles moving in opposite directions result in a transfer of momentum from the parallel direction (x) to the perpendicular direction (y and z). This transition leads to a change in kinetic energy from parallel to perpendicular until reaching equilibrium with an isotropic distribution of kinetic energy components.

The above analysis of $E_{G1}^{x}$ is supported by the statistical velocity distribution function of G_1_ particles in Figure 13. The velocity distribution function of G_1_ particles at time $t_{0}$ in the collision slice corresponds to the blue curve in Figure 13. Since at this time, G_1_ particles are distributed within a wide range of −30 Å to 30 Å in space, including both the pre-shock wave and post-shock wave regions, their velocity distribution function in the shock direction can be approximately regarded as a superposition of the cold Maxwell distribution function in the central region and two high-temperature Maxwell velocity distribution functions on both sides with translational velocities. As the relaxation process progresses, the peak value of the cold Maxwell peak in the central region gradually decreases, corresponding to the heating of the central region by the collision of the shock waves.

Due to particle migration, the energy evolution of G_1_ particles ($E_{G1}$) will show a significant difference from that of particles in the central collision slice ($E_{c}$), and we can discuss this difference to understand the specific impact of migration on the relaxation process. In Figure 11a, the black dashed line shows the change in the average kinetic energy of central slice particles $E_{c}$ over time. Since the central slice has not been affected by the shock waves in the early stages of relaxation and has a lower temperature, its average kinetic energy value at time $t_{0}$ is much smaller than that of G_1_. As the relaxation process progresses, the black dashed curve gradually rises and coincides with the red curve at time $t_{1}$.

In this process, on the one hand, particles within the collision slice increase their energy through collisions with high-energy particles from the central region. On the other hand, particle migration also makes a significant contribution to the increase in average kinetic energy. Here, the role of migration in increasing average kinetic energy $E_{c}$ includes both the contribution of external high-energy particles entering the slice and the contribution of low-temperature particles in the central slice migrating out of the slice. It should be noted that the specific particle migration process exhibits strong randomness. During the relaxation process, particles entering through migration may also be low-energy particles, and those leaving may be high-energy particles, thereby contributing to a decrease in the average particle kinetic energy. However, from a statistical perspective, the average kinetic energy of particles within the collision slice continues to increase during the relaxation process. The contribution of particle migration to the average particle kinetic energy, as obtained through statistical analysis, is also positive.

The change in $E_{G1}$ from $t_{0}$ to $t_{1}$ is approximated as the contribution of particle collisions to the average particle energy change, denoted as $\Delta E_{collision}$. Meanwhile, the change in $E_{c}$ is considered the total change in average particle energy during the relaxation process, denoted as $\Delta E_{total}$. We subtract from the two yields the change caused by migration, denoted as $\Delta E_{migration}$. This relationship can be expressed by the following formulas as

(10) $$\begin{array}{cc} \Delta E_{collision} & =E_{G1}\left(t_{1}\right)-E_{G1}\left(t_{0}\right), \end{array}$$

(11) $$\begin{array}{cc} \Delta E_{total} & =E_{c}\left(t_{1}\right)-E_{c}\left(t_{0}\right), \end{array}$$

(12) $$\begin{array}{cc} \Delta E_{migration} & =\Delta E_{total}-\Delta E_{collision}. \end{array}$$

By dividing the contributions of collision and migration by the total change, we can quantitatively assess the proportion of the contributions of particle migration and collision to the particle kinetic energy during the relaxation process. We systematically calculated the proportions for different $v_{p}$ cases, and the results are summarized in Table 4. It can be observed that the proportion of particle migration effects remains relatively constant with increasing $v_{p}$, consistently exceeding 50%. This indicates that the impact of particle migration on the relaxation process is quite significant.

To quantify the impact of migration and collisions on the relaxation process, we calculated the time derivative of the average kinetic energy and its components, as shown in Figure 11b. The black dashed line represents the rate of energy change $E_{c}$ from both collisions and migrations. Initially, the energy change rate is low but peaks around 1160 fs when the shock waves begin to collide.

The red solid line shows the rate of change in energy due to particle collisions ($E_{G1}$), which remains stable throughout the relaxation period, only dropping to zero in the late phase.

Since the energy from collisions is distributed as either thermal motion kinetic energy or translational kinetic energy, we analyzed how the added energy is allocated between these two forms. Assuming isotropy in the thermal motion components, the thermal kinetic energy of G_1_ particles is approximated as $E_{G1}^{th}=3E_{G1}^{y}$ and the translational kinetic energy as $E_{G1}^{trans}=E_{G1}^{x}-E_{G1}^{y}$. By calculating their rates of change over time, shown in Figure 12, we observe that before 1200 fs, the rates for both energy types are similar, indicating that the additional energy from collisions is proportionally distributed between translational and thermal motion kinetic energy. This behavior aligns with that of a single shock wave in ideal gas [49]. After 1200 fs, the translational kinetic energy decreases as the shock waves begin to collide directly, converting the lost translational energy into thermal energy for G_1_ particles.

## 4. Conclusions

This study systematically investigates the collision process of strong shock waves propagating through a classical helium medium using the Non-Equilibrium Molecular Dynamics (NEMD) method. The simulation results reveal a decrease in relaxation times for various thermodynamic quantities as the shock piston velocity increases, and this relationship is accurately modeled by an inverse proportional function. We extensively discuss the influence of particle migration and collisions on the change in particle kinetic energy during the relaxation process. The findings suggest that particle migration significantly accelerates the relaxation process. Unlike typical relaxation process studies that primarily focus on microscopic particle collision mechanisms, the results of this work indicate that in the relaxation process during strong shock wave collisions, particle migration contributes significantly alongside particle collision mechanisms. This study provides valuable insights into the fundamental physics of shock wave collisions, particularly the discussion on particle migration effects, offering a novel perspective on understanding the evolution of relaxation processes in shock wave collisions.

Considering the substantial simplifications made in this work regarding the strong shock wave collision process, a more accurate quantitative assessment of the impact of particle migration on relaxation processes during strong shock wave collisions in real-world scenarios would require consideration of factors such as electron ionization and the potential function under extreme conditions. Building upon the results of this research, incorporating a deeper understanding of additional complex factors will allow for a more detailed exploration of the contributions of different factors to the relaxation process. This could serve as a basis for future research endeavors.

## Figures and Tables

**Table 1:** The state parameters (density ρ, temperature *T*, and pressure *P*) behind the shock front and shock speed $v_{s}$ of the first and the second shocks under different $v_{p}$.

$v_{p}$	$\rho_{1}$	$T_{1}$	$P_{1}$	$v_{s1}$	$\rho_{2}$	$T_{2}$	$P_{2}$	$v_{s2}$
km/s	g/cm^3^	ev	GPa	km/s	g/cm^3^	ev	GPa	km/s
10	0.396	1.206	21.32	14.8	0.693	2.757	112.1	13.4
20	0.426	4.998	82.32	28.2	0.787	12.06	458.9	23.7
30	0.443	11.20	178.6	42.3	0.850	26.71	1017	32.8
40	0.455	20.68	320.8	55.8	0.886	47.39	1792	41.9
50	0.464	31.88	486.6	68.4	0.921	74.97	2764	50.4
60	0.470	46.53	707.2	82.3	0.946	112.0	4154	59.6
70	0.473	63.77	957.0	96.2	0.964	146.5	5404	67.1
80	0.478	83.59	1243	109	0.973	198.2	7149	76.8
90	0.483	103.4	1544	122	0.998	249.9	9112	83.9
100	0.488	129.3	1920	135	1.01	310.2	11253	93.6

**Table 2:** Calculated relaxation time for various thermodynamic quantities under different $v_{p}$.

$v_{p}$	$\tau_{\rho }$	$\tau_{T}$	$\tau_{T_{x}}$	$\tau_{T_{z}}$
km/s	fs	fs	fs	fs
10	138.80	142.50	191.30	138.30
20	88.30	85.94	115.78	93.07
30	76.24	75.96	101.24	68.40
40	72.08	68.76	90.41	64.15
50	55.41	52.53	70.17	50.69
60	49.39	47.52	58.34	46.45
70	45.23	45.97	52.08	43.24
80	42.13	41.86	49.31	41.67
90	37.62	37.09	43.37	38.98
100	32.77	33.59	39.80	33.45

**Table 3:** Values of fitting parameters for $\tau_{\rho }$, $\tau_{T}$, and $\tau_{Tx}$.

	*a* (fs^−1^)	*b* (Å)	*c* (fs/Å^2^)
$\tau_{\rho }$	$4.47\times 10^{-3}$	$2.97\times 10^{-2}$	$-5.24\times 10^{-7}$
$\tau_{T}$	$4.05\times 10^{-3}$	$3.24\times 10^{-2}$	$-6.60\times 10^{-7}$
$\tau_{T_{x}}$	$2.81\times 10^{-3}$	$2.56\times 10^{-2}$	$-3.89\times 10^{-7}$

**Table 4:** The proportion of contributions from particle migration and collisions to the average particle kinetic energy change during the relaxation process.

$v_{p}$ (km/s)	Collisions (%)	Migration (%)
10	40.2	59.8
20	36.9	63.1
30	38.6	61.4
40	52.5	47.5
50	46.7	53.3
60	40.3	59.7
70	39.5	60.5
80	42.3	57.7
90	34.9	65.1
100	48.6	51.4

## Data Availability

Data are contained within the article.
